# Occurrence and Antimicrobial Resistance of *Salmonella* in Raw Beef and Meat Contact Surfaces: A Cross‐Sectional Study From Hossana Town, Central Ethiopia

**DOI:** 10.1155/cjid/7477794

**Published:** 2026-02-25

**Authors:** Assefa Alemu, Galana Abaya, Girma Godebo, Abdulhakim Mussema

**Affiliations:** ^1^ Department of Biotechnology, Wachemo University, Hossana, Ethiopia, wcu.edu.et; ^2^ Department of Medical Laboratory Science, Wachemo University, Hossana, Ethiopia, wcu.edu.et

**Keywords:** antimicrobial resistance, Ethiopia, foodborne illness, *Salmonella*

## Abstract

**Introduction:**

*Salmonella* is a leading cause of foodborne illness worldwide, with a rising concern for the developing and spreading of antimicrobial‐resistant strains due to the imprudent utilization of antimicrobials. Continuous surveillance of *Salmonella* resistance patterns is critical. This study aims to estimate the occurrence of *Salmonella* species (spp.) in raw beef and on meat contact surfaces in Hossana Town, Central Ethiopia. Additionally, it seeks to identify associated risk factors that contribute to the presence of *Salmonella* and to assess the antimicrobial susceptibility profile of the *Salmonella* isolates.

**Materials and Methods:**

A cross‐sectional study was conducted from May 2023 to December 2023, involving 370 raw beef and meat contact surfaces selected through simple random sampling. Sociodemographic data, hygiene practices of meat handlers, and factors contributing to meat contamination at randomly selected abattoirs and retail outlets were evaluated by semistructured questionnaire and observation checklists. *Salmonella* was isolated and identified by using standard bacteriological culture methods. Antimicrobial susceptibility was evaluated using the Kirby–Bauer disk diffusion method and data were analyzed by using SPSS version 2020, with significance set at *p* < 0.05.

**Results:**

Out of a total of 370 samples that were collected using a simple random sampling method, 31 (8.4%) (95% confidence interval [CI]: 5.2–12.2) were tested positive for *Salmonella* isolates using biochemical tests. Multivariable logistic regression analysis revealed that having less than 1 year of service, lack of food safety and hygiene training, not undergoing medical checkups in the past 6 months, failure to use sanitizer, not washing hands with soap before and after meat processing, absence of protective gear, lack of hygiene of slicing material, and absence of flies control at meat retailer outlets were among the potential risk factors that significantly correlated with the occurrence *Salmonella* spp. in the study area. *Salmonella* isolates were 100% susceptible for both ciprofloxacin and gentamycin, whereas the highest resistance rate (93.5%) was observed against tetracycline.

**Conclusion and Recommendations:**

The study revealed an 8.4% occurrence of *Salmonella* isolates, indicating a serious public health issue driven by key factors like inadequate training, lack of medical check‐ups, poor hand hygiene, insufficient protective clothing, and unsanitary equipment. While the isolates were susceptible to ciprofloxacin and gentamicin, there was a concerning 93.5% resistance to tetracycline, highlighting the need for better antibiotic stewardship in the meat supply chain. To address these issues, it is recommended to implement hygiene training for food handlers, mandatory medical check‐ups, and enforcement of personal protective clothing, regular hygiene audits, and public awareness campaigns to mitigate *Salmonella* risks.

## 1. Introduction

Foodborne diseases (FBDs) are illnesses resulting from the consumption of food contaminated with pathogenic microbes, posing a significant public health issue globally, particularly in developing countries [1]. Many individuals suffer from these illnesses due to contaminated food and water, with *Salmonella* being one of the most common pathogens in animal‐derived foods, leading to thousands of deaths annually [2–5]. It is estimated that *Salmonella* causes approximately 115 million infections and 370,000 deaths worldwide each year, making it the second most reported foodborne gastrointestinal infection after campylobacteriosis (Qin et al., 2022) [6].

The organism *Salmonella* was first recognized by Theobald Smith in 1884, and a year later, he and Daniel Elmer Salmon isolated *Salmonella choleraesuis* from infected pigs [7]. This genus consists of Gram‐negative, facultative anaerobic, non‐spore‐forming bacilli within the Enterobacteriaceae family, with over 2700 serotypes classified into three species: *Salmonella bongori*, *S. subterranean*, and *S. enterica* [8, 9]. Most disease‐causing isolates belong to *S. enterica*, which is further divided into over 2600 serovars that thrive in the digestive tracts of various food‐producing animals.

In Ethiopia, FBDs are prevalent due to poor food handling practices, inadequate sanitation, weak regulatory systems, and a lack of education among food handlers [2, 10, 11]. *Salmonella* is one of the most common pathogens in the country and a major cause of foodborne illnesses [12, 13]. The virulence factors of *Salmonella* play a crucial role in host infection and disease spread, primarily located in chromosomal genes known as *Salmonella* pathogenicity islands [14].

Meat is a crucial source of high‐quality protein, B vitamins, and essential minerals, significantly contributing to bodily functions and daily activities [15, 16]. However, its nutrient‐rich composition also creates an ideal environment for pathogenic bacteria. Beef, being the third most consumed meat globally, poses particular risks [17]. In Ethiopia, the traditional practice of consuming raw or undercooked meat heightens the risk of foodborne illnesses, particularly from *Salmonella*, which can contaminate beef at multiple stages from slaughter to retail [18, 19]. Therefore, evaluating meat safety at these critical points is essential for understanding consumer exposure to enteric pathogens and the associated risk of antimicrobial resistance (AMR) [19].

The rise of antibiotic resistance (AR) presents a significant public health threat, primarily driven by the widespread use of antibiotics in agriculture for growth promotion and disease prevention in livestock [20]. This practice has contributed to the emergence of multidrug‐resistant (MDR) strains of *Salmonella* that are resistant to essential antimicrobials, including third‐generation cephalosporins and fluoroquinolones [21, 22]. While gastrointestinal salmonellosis often resolves without antibiotic treatment, severe cases require immediate intervention, particularly for vulnerable populations such as children and the elderly [23]. The misuse and overuse of antimicrobial agents in both human and veterinary medicine exacerbate the alarming spread of these resistant bacteria through the food supply chain [24, 25].

FBDs have emerged as a major public health and economic concern in the 21st century, affecting about one‐third of the population in developed countries each year and resulting in billions of dollars in healthcare and social costs [26–28]. The incidence of FBDs is increasing globally, particularly in developing nations like Ethiopia, where poor food handling practices, inadequate sanitation, and weak regulatory frameworks prevail. Approximately two million people die each year in developing countries due to foodborne pathogens [24].

AMR is a pressing global issue that significantly impacts public health. It is primarily attributed to the misuse of antibiotics in livestock [29]. Multidrug‐resistant *Salmonella* poses a substantial public health risk as contaminated food can transmit resistant strains to humans, potentially leading to the accumulation of resistance genes in the human gut microbiota [30, 31]. The latest report examines 208,233 *Salmonella* genomes collected from 148 countries between 1900 and 2023 to evaluate patterns of AMR. It reveals that chicken‐related serovars and those from environmental sources exhibit higher resistance levels, whereas serovars associated with cattle and pigs are experiencing a decline. The study identifies several factors affecting AMR, including antibiotic usage, agricultural practices, climate conditions, and socioeconomic factors, contributing to the development of a genetic atlas for improved comprehension [32]. Currently, AMR is estimated to cause around 700,000 deaths annually, with projections suggesting this could rise to 10 million by 2050, resulting in economic losses reaching $100 trillion [8].

In Ethiopia, various studies have studied the prevalence and antimicrobial susceptibility of *Salmonella* in raw beef [11, 33–36]. Additionally, *Salmonella* isolates exhibited varying occurrence rates and a high level of drug resistance to commonly used antibiotics in the current study area of Hossana Town, obtained from stool specimens of both symptomatic and asymptomatic individuals [37, 38].

While extensive research has been conducted on *Salmonella* in various food products and other specimens, significant gaps persist, particularly concerning its occurrence and AMR in raw beef and meat contact surfaces in Hossana town. Although some studies have documented the presence of *Salmonella* in raw meat, there is also a notable lack of data regarding contaminations of meat contact surfaces within local markets and processing facilities. Understanding these contamination points is vital for evaluating food safety risks.

Moreover, limited research has focused on how local meat handling practices influence the occurrence of *Salmonella*, which is essential for developing effective food safety interventions. To address these gaps, this study aims to assess both the occurrence and AMR of *Salmonella* in raw beef and on meat contact surfaces in Hossana town’s municipal abattoir and retail outlets. The findings will contribute to improving food safety measures and public health strategies in the study area.

## 2. Materials and Methods

### 2.1. Description of Study Area

**FIGURE 1:**
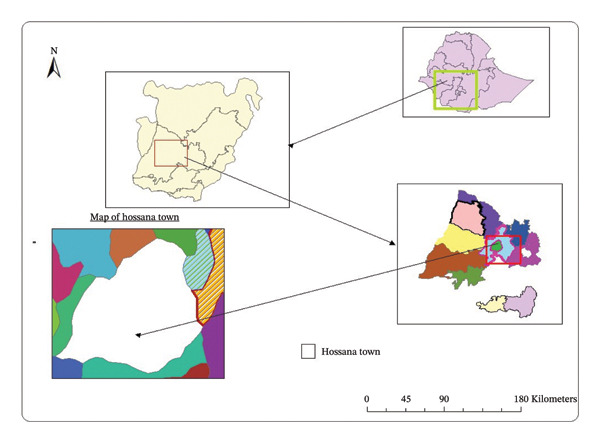
Map of the study area (HTAO, 2023).

The study was conducted in the Hossana Town Administration, Hadiya Zone, Central Ethiopia (Figure 1). Hossana Town is located at a distance of 232 km south of Addis Ababa via the main road that connects Alemgena, Butajera, and Sodo towns. The distances from Addis Ababa via the Wolkite and Zeway roads are around 280 and 305 km, respectively. Geographically, it is located at a latitude of 7.58 (7°34′ 60″ N) and a longitude of 37.88 (37°52′ 60″ E), and the altitude of the district ranges from 1600 to 2240 m above sea level. It has a bimodal rainfall pattern (long and short rainy seasons). The short rainy season runs from March to April, whereas the long rainy season lasts from June to September. The average annual rainfall is between 950 and 1200 mm, and the maximum and minimum temperatures are 23°C and 13°C, respectively [39].

### 2.2. Study Samples

The study samples were sourced from seemingly healthy young male bovines (bulls and oxen) brought from various regions of central Ethiopia for slaughter at the municipal abattoir in Hossana Town, as well as from meat handlers at the abattoir and retail outlets. Bovine slaughter that exhibited clinical signs of salmonellosis, along with meat handlers who had communication difficulties due to disabilities or illnesses, and severely ill workers, were excluded from the study.

### 2.3. Study Design

A cross‐sectional study was conducted from May 2023 to December 2023 to determine the occurrence, associated risk factors, and antimicrobial susceptibility profiles of *Salmonella species* from raw beef and meat contact surfaces in Hossana town.

### 2.4. Sample Size Determination and Sampling Techniques

According to the statistical formula given by Thrusfield [40], the sample size (*n*) was determined by taking 12.5% of the overall prevalence obtained from raw beef and swab samples at the Wolaita Sodo municipal abattoir reported by Wabeto et al. [11], with the assumptions of standard normal deviations (*Z*_*α*/2_ = 1.96) at a 95% confidence interval (CI) and an absolute precision (d) of 0.05% and 10% for the nonresponse rate.

(1) n=Zα/22∗Ppre 1−Ppred2.

The required sample size (*n*) was determined using the formula where *Z*_*α*/2_ is the standard normal deviation (1.96) at a 95% confidence level, Ppre is the preceding prevalence, and *d* is the desired absolute precision. The minimum sample size calculated was 185, accounting for a 10% nonresponse rate, but it was doubled for greater precision, resulting in a total of 370 meat and swab samples from contact surfaces. A simple random sampling method was used to select bulls/oxen at the Hossana municipal abattoir and beef/swab samples from retail outlets. Ultimately, 173 samples (110 beef and 63 swabs) were collected from the abattoir and 197 samples (91 beef and 106 swabs) were collected from retail outlets.

### 2.5. Methods of Data Collection

Sociodemographic data, hygiene practices of meat handlers, and factors contributing to meat contamination were evaluated using a semi‐structured questionnaire and observation checklists. The questionnaire, adapted from prior research [10, 41–43], included three sections: (I) sociodemographic information of meat handlers; (II) knowledge of food safety and hygiene; and (III) potential risk factors for *Salmonella* contamination in meat and contact surfaces. Respondents were randomly selected, primarily from meat retailers due to the limited number of abattoirs in the area. Bulls or oxen were chosen randomly during ante‐mortem inspections, and samples were collected from both abattoir and retail outlets. Workers at these locations were also randomly selected for interviews, and with their consent, meat and swab samples were collected.

### 2.6. Sample Collection and Transportation

Meat and contact surface samples were collected and transported according to ISO‐17604 guideline [44]. Fresh meat samples were taken immediately after processing in the abattoir and within 3 hours of arrival at retail outlets. Sampling focused on areas prone to contamination during slaughter, including the rump, flank, brisket, and neck, using sterile aluminum foil templates (10 × 10 cm). A sterile cotton swab, presoaked in buffered peptone water (BPW), was used to swab the designated areas both horizontally and vertically. Approximately 25 g of fresh beef was then placed in a sterile bottle with BPW, with the swab left inside after breaking the shaft.

In addition, swabs were taken from meat contact surfaces such as knives, handlers’ hands, and hooks, axes, cutting boards, and mincing machines, using BPW‐moistened cotton swabs. All samples were labeled with collection details and transported in an ice box to Wachemo University Medical Microbiology Laboratory within 4 hours for bacteriological analysis, phenotypic characterization, and antimicrobial susceptibility testing (AST). Upon arrival, samples were refrigerated at 4°C until analysis if there is delayed before processing.

### 2.7. Bacteriological Isolation of *Salmonella*

#### 2.7.1. Pre‐Enrichment in Nonselective Broth

*Salmonella* was isolated according to the microbiology of the food chain; horizontal methods for the detection of *Salmonella* [44]. The preparations of meat and swab samples, and the routine isolation of *Salmonella* in the laboratory, were carried out in accordance with the procedures of the International Organization for Standardization [44]. The swab samples were pre‐enriched with an appropriate amount of BPW and incubated at 37°C for 24 h. Briefly, 25 g of each beef sample was weighed, cut into small pieces with different sterile scalpel surgical blades, and transferred aseptically into a sterile conical flask containing 225 mL of sterile BPW as a pre‐enrichment medium, then homogenized by using a vortex mixer for 2 min. The pre‐enriched samples were then incubated at 37°C for 18–24 h [44].

#### 2.7.2. Enrichment in Selective Broth Media

Selective enrichment media, namely Rappaport‐Vassiliadis Soy (RVS) broth (Himedia, India‐M880‐500G), was used for all beef and swap samples to inhibit microorganisms such as nontarget Gram‐positive bacteria and coliforms, and allow rapid duplication of *Salmonella.* After pre‐enrichment in BPW, 0.1 mL (100 μL) of the pre‐enrichment culture was transferred aseptically with the help of a sterile micro‐pipette into 10 mL of RVS broths and incubated at 42°C for 24 h [44].

#### 2.7.3. Selective Plating Out and *Isolation*

The selective solid media, Salmonella‐Shigella Agar (SSA) (Accumix®, India‐AM5093‐500G) plates were used for plating and isolation purposes. A loop full of inoculums from RVS broth was transferred and streaked onto the surface of SSA plates, and the plates were incubated aerobically at 37°C for 18–24 h. After incubation, the plates were examined for the presence of characteristics associated with *Salmonella* colonies. A single positive colony showing characteristics of *Salmonella* colonies on SSA was subcultured on nutrient agar (NA) (HKM‐HCM007‐500G) and incubated at 37°C for 18–24 h. Further, a discrete colony from NA was subcultured on nutrient broth (NB) (Accumix®, India‐AM5077‐500G) and incubated at 37°C for 18–24 h. Thereafter, the suspected isolates of *Salmonella* spp. were preserved in 30% bacterial glycerol stock and kept at −20°C until biochemical characterization [44].

#### 2.7.4. Biochemical Characterization of Suspected Isolates of *Salmonella* Species

The biochemical characterization of the suspected *Salmonella* spp. was performed by different biochemical tests, and the results were interpreted according to the guidelines of the International Organization for Standardization [44]. Bacterial isolates from glycerol stock were refreshed on NA, and their biochemical characteristics were determined by using a triple sugar iron agar (TSIA) test (HKM, China‐HCM014‐500G), decarboxylase Test (HIMEDIA, India, M912‐500G), a motility test (Accumix®, India, 201130590‐500G), sulfide, indole, motility (SIM) test (HKM, China‐026051‐500G), and the urease test (Oxoid, England, CM0053‐500G). A brief interpretation of the biochemical tests used for identifying *Salmonella* isolates and related bacterial isolates is provided below (Table 1).

**TABLE 1:** Results of biochemical tests for *Salmonella* and other bacteria with similar biochemical characters.

Biochemical tests	*Salmonella* spp.	*Citrobacter freundii*	*Citrobacter diversus/amalonaticus*	*Shigella* spp.	*Proteus* spp.	*Hafnia* alvei
Lactose fermentation	−	±	±	−	−	−
Indole production	−	−	+	±	±	−
H2S production in TSI	+	±	−	−	±	−
Lysine decarboxylation	+	−	−	±	−	+
Motility	+	+	+	−	+	+
Urease production	−	±	±	−	±	±

*Note:* + = positive reactions, − = negative reactions, and ± = different reactions according to the strains.

#### 2.7.5. AST

AST of phenotypically characterized *Salmonella* isolates was performed using the Kirby–Bauer disk diffusion method on Muller‐Hinton agar, following Hudzicki [45] and interpreted according to Clinical and Laboratory Standard Institute (CLSI) (2020) guideline. Antibiotics were chosen due to their importance to public health and their frequent use in treating *Salmonella* infections in Ethiopia, in accordance with the CLSI [46] guideline. This selection includes eight antimicrobials from seven different classes: ampicillin, cefixime, ciprofloxacin, ceftazidime, amoxicillin‐clavulanic acid, tetracycline, gentamicin, and co‐trimoxazole. After incubation, the inhibition zones were measured to determine sensitivity (*S*), intermediate resistance (*I*), or resistance (*R*). Isolates resistant to three or more antibiotic classes were classified as MDR [47] (Table 2).

**TABLE 2:** Performance standard of antibiotic discs for antimicrobial susceptibility testing of *Salmonella* spp.

Antimicrobial classes	Antimicrobial agents	Disc code	Potency (μg)	Interpretive categories and zone diameter breakpoints in mm		
				*R* ≤	*I*	*S* ≥
Penicillin	Ampicillin	AMP	10	13	14–16	17
Quinolones	Ciprofloxacin	CPR	5	20	21–30	31
Cephalosporins	Ceftazidime Cefixime	CAZ CXM	30 5	17 15	18–20 16–18	21 19
B‐lactam combination agent	Amoxycillin‐Clavulanic Acid	AMC	3	13	14–17	18
Tetracycline	Tetracycline	TE	30	11	12–14	15
Folate‐pathway antagonist	Co‐trimoxazole	COT	25	10	11–15	16
Aminoglycoside	Gentamicin	CN	10	12	13–14	15

*Note: Source:* [46].

#### 2.7.6. Laboratory Quality Control (QC)

The reliability of the study findings was ensured by implementing QC measures throughout the laboratory process. All materials, equipment, and procedures were rigorously monitored, and each procedure was conducted aseptically. The quality of the culture media, Gram stain, and antimicrobial discs was verified using standardized reference strains of *Salmonella typhimurium* ATCC 14028 and *Escherichia coli* ATCC 25922, in accordance with CLSI guidelines. To standardize the inoculum density for the susceptibility test, a turbidity standard equivalent to a 0.5 McFarland standard was utilized according to the CLSI [46] guideline.

#### 2.7.7. Data Management and Statistical Analysis

The study’s data, including laboratory findings, underwent a thorough verification, coding, and organization process. Statistical analyses were conducted using SPSS Version 2020, beginning with descriptive analysis to assess sociodemographic distributions. To explore the relationships between independent and dependent variables, both bivariate and multivariable logistic regression analyses were employed. Initially, univariable logistic regression was performed to calculate crude odds ratios and associated *p* values for the identified associations. Independent variables that yielded *p* values below 0.25 were subsequently analyzed in a multivariable model to control for potential confounders and pinpoint significant risk factors. The evaluation of significant associations was based on adjusted odds ratios (AOR) along with 95% CI and *p* values, with a significance level set at *p* < 0.05.

## 3. Results

### 3.1. Sociodemographic Characteristics of Meat Handlers

According to the sociodemographic data collected from workers at abattoir and meat retailer outlets in the Hossana town, 85.3% of the respondents were men, and 39.74% of them were in the 26–35 age range. Of the participants, the workers with secondary education made up the majority (38.46%). The majority (36.54%) of the workers had work experience between 3 and 5 years. On the other hand, the majority of employees (51.92%) at meat retailer outlets and abattoir earned less than 3000 birr per month, and 63.46% of respondents had contract employment status (Table 3).

**TABLE 3:** Sociodemographic characteristics of meat handlers working in abattoir and meat retailer outlets in Hossana town (*n* = 156).

Variables	Categories	Meat handlers		Total sample tested	Positive *n* (%)
		Freq.	Per. (%)		
Gender	Male	133	85.3	338	27 (7.9)
	Female	23	14.7	32	4 (12.5)

Age	≤ 25	39	25	80	9 (11.25)
	26–35	62	39.74	164	14 (8.5)
	36–45	34	21.79	70	3 (4.3)
	Above 45	21	13.46	56	5 (8.9)

Marital status	Single	94	60.26	239	17 (7.1)
	Married	58	37.18	121	13 (10.1)
	Divorced	4	2.56	10	1 (10.0)

Level of education	Unable to read and write	10	6.41	19	3 (15.79)
	Informal education	15	9.62	33	4 (12.12)
	Primary education	46	29.48	121	14 (11.1)
	Secondary education	60	38.46	158	8 (5.06)
	College and above	25	16.03	39	2 (5.13)

Year of service	Less than 1 year	21	13.46	41	5 (12.2)
	1–2 years	45	28.85	112	12 (10.7)
	3–5 years	57	36.54	142	11 (7.7)
	More than 5 years	33	21.15	75	3 (4.0)

Salary in ETB	Less than 3000	81	51.92	194	20 (10.3)
	3001–6000	45	28.85	104	8 (7.7)
	Above 6000	30	19.23	72	3 (4.2)

Employment status	Contract	99	63.46	167	12 (7.2)
	Permanent	57	36.54	203	19 (9.4)

*Note:* Freq. = frequency, Per. = percent, and ETB = Ethiopian Birr.

### 3.2. Isolation and Occurrence of *Salmonella*

A total of 57 *Salmonella* suspected colorless/transparent colonies with or without black centers Salmonella‐Shigella Agar were obtained from 370 raw beef and swab samples. Among the 57 presumptively identified isolates of *Salmonella* spp., 31 (8.4%) (95% CI: 5.2–12.2) isolates showed typical characteristics of *Salmonella* spp. based on various biochemical tests. Out of them, 12 (6.9%) and 19 (9.6%) were isolated from slaughterhouse and meat retailer outlets, respectively (Table 4).

**TABLE 4:** Occurrence of *Salmonella* spp. from abattoir and meat retailer outlets in Hossana town from May to December, 2023.

Source of sample	Type of sample		No. of samples examined	Status of PIISSs (*n* (%))		*χ* ^2^ (*p*value)
				Negative	Positive	
Abattoir	Meat	Rump	31	30 (96.8)	1 (3.2)	0.88 (0.348)
		Flank	28	26 (92.9)	2 (7.1)	
		Brisket	21	19 (90.5)	2 (9.5)	
		Neck	30	29 (96.7)	1 (3.3)	
		Subtotal	110	104 (94.6)	6 (5.4)	
	Swab	Hand	15	13 (86.7)	2 (13.3)	
		Knife	19	17 (89.5)	2 (10.5)	
		Hook	12	12 (100)	0 (0)	
		Axe/saw	17	15 (88.2)	2 (11.8)	
		Subtotal	63	57 (90.5)	6 (9.5)	
		Total	173	161 (93.1)	12 (6.9)	
Meat retailer outlets	Meat	Rump	23	21 (91.3)	2 (8.7)	
		Flank	20	19 (95.0)	1 (5.0)	
		Brisket	23	22 (95.7)	1 (4.3)	
		Neck	25	21 (84.0)	4 (16.0)	
		Subtotal	91	83 (91.2)	8 (8.8)	
	Swab	Hand	14	11 (78.6)	3 (21.4)	
		Knife	20	19 (95.0)	1 (5.0)	
		Hook	11	11 (100)	0 (0)	
		Axe/saw	11	10 (90.9)	1 (9.1)	
		Cutting board	27	23 (85.2)	4 (14.8)	
		Meat mincing machine	23	21 (91.3)	2 (8.7)	
		Subtotal	106	95 (89.6)	11 (10.4)	
		Total	197	178 (90.4)	19 (9.6)	
Overall total			370	339 (91.6%)	31 (8.4%)	

Abbreviation: PIISSs: presumptively identified isolates of Salmonella species

### 3.3. Factors Associated With the Occurrence of *Salmonella* Species

#### 3.3.1. Potential Risk Factors Associated With Beef Contamination

In univariable logistic regression, several factors were significantly associated with *Salmonella* spp. occurrence (*p* ≤ 0.25): education level (COR = 3.5), years of service (COR = 3.3), salary of meat handlers (COR = 2.64), food safety knowledge (COR = 9.7), food safety training (COR = 4.2), recent medical checkups (COR = 17.9), hand sanitizer use (COR = 3.3), handwashing (COR = 3.9), protective gear use (COR = 4.7), shared equipment for offal and meat (COR = 3.2), money handling during sales (COR = 11.1), hygiene of slicing materials (COR = 23.1), cutting board hygiene (COR = 12.3), and fly control measures (COR = 6.9) (Tables 5, 6, 7).

**TABLE 5:** Univariable logistic regression analysis of sociodemographic characteristics of meat handlers working in abattoir and meat retailer outlets in Hossana town (*n* = 156).

Variables	Categories	Meat handlers		Total sample tested	Positive *n* (%)	COR	*p* value	95% CI
		Freq.	Per. (%)					
Gender	Male	133	85.3	338	27 (7.9)	0.38	0.608	0.19–1.86
	Female	23	14.7	32	4 (12.5)	*R*		

Age	≤ 25	39	25	80	9 (11.25)	1.29	0.662	0.41–4.09
	26–35	62	39.74	164	14 (8.5)	0.95	0.928	0.33–2.77
	36–45	34	21.79	70	3 (4.3)	0.46	0.298	0.10–2.00
	Above 45	21	13.46	56	5 (8.9)	*R*		

Marital status	Single	94	60.26	239	17 (7.1)	0.69	0.731	0.08–5.77
	Married	58	37.18	121	13 (10.1)	1.08	0.942	0.127–9.25
	Divorced	4	2.56	10	1 (10.0)	*R*		

Level of education	Unable to read and write	10	6.41	19	3 (15.79)	3.5	0.195^∗^	0.53–22.79
	Informal education	15	9.62	33	4 (12.12)	2.6	0.298	0.44–14.91
	Primary education	46	29.48	121	14 (11.1)	2.4	0.257	0.53–11.16
	Secondary education	60	38.46	158	8 (5.06)	0.9	0.987	0.20–4.84
	College and above	25	16.03	39	2 (5.13)	*R*		

Year of service	Less than 1 year	21	13.46	41	5 (12.2)	3.3	0.112^∗^	0.75–14.73
	1–2 years	45	28.85	112	12 (10.7)	2.9	0.111^∗^	0.78–10.58
	3–5 years	57	36.54	142	11 (7.7)	2.0	0.294	0.55–7.46
	More than 5 years	33	21.15	75	3 (4.0)	*R*		

Salary in ETB	Less than 3000	81	51.92	194	20 (10.3)	2.64	0.126^∗^	0.76–9.18
	3001–6000	45	28.85	104	8 (7.7)	1.92	0.349	0.49–7.49
	Above 6000	30	19.23	72	3 (4.2)	*R*		

Employment status	Contract	99	63.46	167	12 (7.2)	0.698	0.350	0.329–1.48
	Permanent	57	36.54	203	19 (9.4)	*R*		

*Note: R* = reference, Freq. = frequency, Per. = percent, and ETB = Ethiopian Birr.

Abbreviations: CI = confidence interval and COR = crude odds ratio.

^∗^Candidate variables with *p* ≤ 0.25.

**TABLE 6:** Univariable logistic regression analysis of factors associated with meat contamination with the isolates of *Salmonella* spp. in both abattoir and meat retailer outlets.

Variables	Categories	Tested	Positive *n* (%)	COR	*p* value	95% CI
Knows food safety and hygiene	Yes	243	6 (2.5)	*R*		
	No	127	25 (19.7)	9.7	**0.000** ^∗^	3.85–24.31

Food safety training	Yes	248	11 (4.4)	*R*		
	No	122	20 (16.4)	4.2	**0.000** ^∗^	1.95–9.14

Medical checkups	Yes	250	4 (1.6)	*R*		
	No	120	27 (22.5)	17.9	**0.000** ^∗^	6.08–52.41

Hygiene of food handlers	Poor	133	25 (18.8)	9.03	**0.034** ^∗^	1.18–68.88
	Fair	197	5 (2.5)	1.016	0.989	0.12–8.94
	Good	40	1 (2.5)	*R*		

Use of sanitizer	Yes	203	9 (4.4)	*R*		
	No	167	22 (13.2)	3.3	**0.004** ^∗^	1.46–7.31

Process meat during illness	Yes	78	5 (6.4)	0.701	0.482	0.26–1.89
	No	292	26 (8.9)	*R*		

Wash hands with soap	Yes	263	13 (4.9)	*R*		
	No	107	18 (16.8)	3.9	**0.000** ^∗^	1.83–8.26

Use of protective materials	Yes	255	11 (4.3)	*R*		
	No	115	20 (17.4)	4.7	**0.000** ^∗^	2.15–10.11

Use of the same equipment for offal and meat processing	Yes	131	19 (14.5)	3.2	**0.003** ^∗^	1.50–6.84
	No	239	12 (5.0)	*R*		

Remove your work equipment during toilet	Yes	273	22 (8.1)	*R*		
	No	97	9 (9.3)	1.2	0.710	0.51–2.63

*Note: R* = reference.

Abbreviations: CI = confidence interval and COR = crude odds ratio.

^∗^Candidate variables with *p* ≤ 0.25.

**TABLE 7:** Univariable logistic regression analysis of factors associated with meat contamination with the isolates of *Salmonella* spp. in Hossana town at meat retailer outlets.

Variables	Categories	Total sample tested	Positive *n* (%)	COR	*p* value	95% CI
Get visit from health authority	Yes	104	9 (8.7)	*R*		
	No	93	10 (10.8)	1.3	0.619	0.49–3.28

Have different meat storage cabinets	Yes	61	6 (9.8)	*R*		
	No	136	13 (9.6)	0.9	0.951	0.35–2.68

Clean the meat storage properly	Yes	135	12 (8.9)	*R*		
	No	62	7 (11.3)	1.3	0.597	0.49–3.49

Collect money during meat selling	Yes	128	18 (14.1)	11.1	0.020^∗^	1.45–85.25
	No	69	1 (1.4)	*R*		

Hygiene of slicing material	Poor	40	11 (27.5)	23.1	0.003^∗^	2.85–187.88
	Fair	95	7 (7.4)	4.85	0.144^∗^	0.58–40.45
	Good	62	1 (1.6)	*R*		

Hygiene of cutting board	Poor	38	8 (21.1)	12.3	0.021^∗^	1.46–103.13
	Fair	112	10 (8.9)	4.5	0.157^∗^	0.56–36.28
	Good	47	1 (2.1)	*R*		

Use of refrigerator	Yes	116	13 (11.2)	*R*		
	No	81	6 (7.4)	0.6	0.377	0.23–1.74

Presence of flies control	Yes	82	2 (2.4)	*R*		
	No	115	17 (14.8)	6.9	0.011^∗^	1.56–30.93

Presence of ventilator	Yes	110	11 (10.0)	*R*		
	No	87	8 (9.2)	0.9	0.849	0.35 2.37

*Note: R* = reference.

Abbreviations: CI = confidence interval and COR = crude odds ratio.

^∗^Candidate variables with *p* ≤ 0.25.

The multivariable logistic regression analysis revealed several significant factors linked to the presence of *Salmonella* spp. in abattoirs and meat retail establishments. These factors include having less than 1 year of service (AOR = 26.386, *p* ≤ 0.015), lack of food safety and hygiene training (AOR = 5.993, *p* ≤ 0.002), not undergoing medical checkups in the past 6 months (AOR = 15.054, *p* ≤ 0.001), failure to use sanitizer (AOR = 4.290, *p* ≤ 0.012), not washing hands with soap before and after meat processing (AOR = 4.769, *p* ≤ 0.007), absence of protective gear (AOR = 6.045, *p* ≤ 0.002), and using the same equipment for processing offal and meat (AOR = 3.609, *p* ≤ 0.025). All associations were statistically significant at *p* ≤ 0.05 (Table 8).

**TABLE 8:** Multivariable logistic regression analysis of factors associated with meat contamination with the isolates of *Salmonella* spp. at abattoir and meat retailer outlets.

Variables	Categories	Tested	Positive *n* (%)	AOR	*p* value	95% CI
Level of education	Unable to read and write	19	3 (15.8)	4.63	0.285	0.28–76.75
	Informal education	33	4 (12.1)	2.294	0.523	0.18–29.27
	Primary education	121	14 (11.6)	5.419	0.118	0.65–45.05
	Secondary education	158	8 (5.1)	2.421	0.396	0.31–18.64
	College and above	39	2 (5.1)	*R*		

Year of service	Less than 1 year	41	5 (12.2)	26.39	0.015^∗^	1.87–373.0
	1–2 years	112	10 (8.9)	24.14	0.010^∗^	2.11–275.6
	3–5 years	142	13 (9.2)	18.58	0.013^∗^	1.8–186.8
	More than 5 years	75	3 (4.0)	*R*		

Salary in ETB	Less than 3000	194	20 (10.3)	1.377	0.712	0.25–7.53
	3001–6000	104	8 (7.7)	2.827	0.312	0.38–21.19
	Above 6000	72	3 (4.2)	*R*		

Knows food safety and hygiene	Yes	243	6 (2.5)	*R*		
	No	127	25 (19.7)	3.236	0.116	0.75–13.99

Training on food safety and hygiene	Yes	248	11 (4.4)	*R*		
	No	122	20 (16.4)	5.993	0.002^∗^	1.96–18.36

Medical checkups	Yes	250	4 (1.6)	*R*		
	No	120	27 (22.5)	15.054	0.001^∗^	3.02–74.99

Hygiene of food handlers	Poor	133	25 (18.8)	0.614	0.482	0.028–8.26
	Fair	197	5 (2.5)	0.387	0.315	0.023–4.32
	Good	40	1 (2.5)	*R*		

Use of sanitizer	Yes	203	9 (4.4)	*R*		
	No	167	22 (13.2)	4.290	0.012^∗^	1.38–13.34

Wash hands with soap	Yes	263	13 (4.9)	*R*		
	No	107	18 (16.8)	4.769	0.007^∗^	1.54–14.74

Use of protective clothes	Yes	255	11 (4.3)	*R*		
	No	115	20 (17.4)	6.045	0.002^∗^	1.98–18.45

Use of the same equipment for offal and meat processing	Yes	131	19 (14.5)	3.609	0.025^∗^	1.18–11.08
	No	239	12 (5.0)	*R*		

*Note: R* = reference.

Abbreviations: AOR = adjusted odds ratio and CI = confidence interval.

^∗^Variables correlated with occurrence of *Salmonella* spp. at *p* ≤ 0.05.

In addition, the multivariable logistic regression analysis revealed significant factors associated to the presence of *Salmonella* spp. at meat retailer outlets. These include collect money during meat selling (AOR = 8.9, *p* ≤ 0.040), lack of hygiene of slicing material (AOR = 13.9, *p* ≤ 0.034), and absence of flies control at meat retailer outlets (AOR = 5.9, *p* ≤ 0.025) were significantly associated with the occurrence of *Salmonella* isolates (Table 9).

**TABLE 9:** Multivariable logistic regression analysis of factors associated with meat contamination with isolates of *Salmonella* spp. in Hossana town at meat retailer outlets.

Variables	Categories	Total sample tested	Positive *n* (%)	AOR	*p* value	95% CI
Collect money during meat selling	Yes	128	18 (14.1)	8.9	0.040^∗^	1.10–71.25
	No	69	1 (1.4)	*R*		

Hygiene of slicing material	Poor	40	11 (27.5)	13.9	0.034^∗^	1.21–159.99
	Fair	95	7 (7.4)	3.1	0.333	0.316–30.09
	Good	62	1 (1.6)	*R*		

Hygiene of cutting board	Poor	38	8 (21.1)	2.4	0.510	0.18–29.97
	Fair	112	10 (8.9)	2.6	0.419	0.26–26.03
	Good	47	1 (2.1)	*R*		

Presence of flies control	Yes	82	2 (2.4)	*R*		
	No	115	17 (14.8)	5.9	0.025^∗^	1.25–28.26

*Note: R* = reference.

Abbreviations: AOR = adjusted odds ratio and CI = confidence interval.

^∗^Variables correlated with occurrence of *Salmonella* at *p* ≤ 0.05.

#### 3.3.2. AST

**FIGURE 2:**
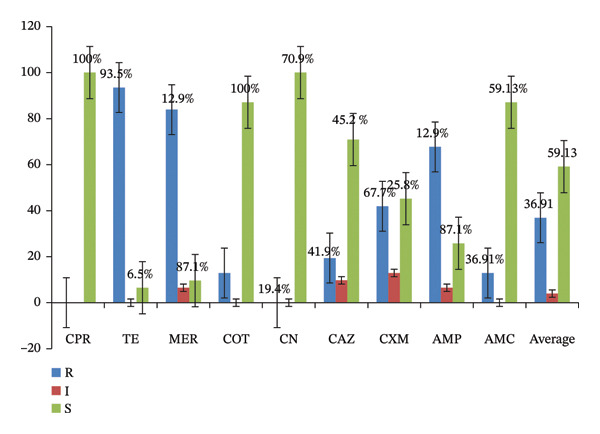
Antimicrobial susceptibility test results of isolates of *Salmonella* spp. CPR = ciprofloxacin, TE = tetracycline, COT = co‐trimoxazole, CN = gentamicin, CAZ = ceftazidime, CXM = cefixime, AMP = ampicillin, AMC = amoxycillin‐clavulanic acid, *R* = resistant, *I* = intermediate, and *S* = susceptible.

The in vitro antibiotic sensitivity tests for *Salmonella* spp. isolates showed varying sensitivity levels from 6.5% to 100%. Among the 31 isolates, 93.5%, 67.7%, and 41.9% exhibited resistance to tetracycline, ampicillin, and cefixime, respectively. In contrast, ciprofloxacin and gentamicin demonstrated full susceptibility (100%), with amoxicillin‐clavulanic acid, co‐trimoxazole, and ceftazidime showing sensitivities of 87.1%, 83.9%, and 70.9%, respectively (Figure 2).

The isolates that exhibited resistance to three or more different classes of antimicrobial agents were regarded as MDR [48]. In this investigation, all of the 31 isolates of *Salmonella* spp. (100%) were resistant to at least one antibiotic, and 25 isolates showed multidrug resistance, yielding a high rate of 80.65% ((Table 10).

**TABLE 10:** Overall antibiotic resistance profiles of isolates of *Salmonella spp. showing* multidrug resistance and multiple antibiotic resistance (MAR) indices.

Antimicrobial resistance patterns	No. of antimicrobial resistance	No. of antimicrobial classes	No. of isolates
TE	1	1	1
CXM‐AMP	2	2	1
TE‐AMP	2	2	1
TE‐CAZ	2	2	1
TE‐COT	2	2	1
TE‐CXM	2	2	1
TE‐CAZ‐AMP	3	3	4
TE‐CXM‐AMP	3	4	5
TE‐COT‐CXM	3	4	4
TE‐AMP‐CXM‐AMC	4	5	5
TE‐CAZ‐CXM‐AMP	4	4	2
TE‐COT‐CAZ‐AMP	4	5	2
TE‐AMP‐CXM‐AMC‐CAZ	5	5	2
TE‐ COT‐CAZ‐CXM‐AMP‐AMC	6	6	1

*Note:* CPR = ciprofloxacin, TE = tetracycline, COT = co‐trimoxazole, CN = gentamicin, CAZ = ceftazidime, CXM = cefixime, AMP = ampicillin, and AMC = amoxycillin‐clavulanic acid.

## 4. Discussion

The study found an overall *Salmonella* spp. occurrence of 8.4% (95% CI: 5.2–12.2) using conventional culture methods, comparable to previous studies in Jimma, Ethiopia (11.3%) by Takele et al. [49], Saudi Arabia (8.5%) by Bahnass et al. [50], and Central India (6%) by Kalambhe et al. [51], but higher than those studies in Dire Dawa (2.75%) by Mengistu et al. [52], Addis Abeba (3.7%) by Ketema et al. [53], and Hawassa (4.1%) by Worku et al. [19]. In contrast, studies from Ghana (30%) by Ekli et al. [54] and Wolaita Sodo (12.5%) by Wabeto et al. [11] reported higher occurrence rates. Supporting these findings, a recent analysis of 208,233 *Salmonella* genomes from 148 countries between 1900 and 2023 highlights that AMR levels also vary geographically, influenced by factors such as location, source, and serovar [32].

The observed differences in *Salmonella* spp. occurrence, with 6.9% in abattoirs and 9.6% in retail outlets, may be primarily attributed to variations in sanitation and hygiene practices between these environments; specifically, the higher contamination rates on contact surfaces—54.8% overall, with the most significant contamination found in hand swabs (21.4%) and cutting boards (14.8%) at retail outlets—suggest poor hygiene practices during meat handling in retail settings contribute significantly to the increased risk of *Salmonella* presence [33].

Of the 31 isolates, 14/31 (45.2%) came from raw beef, with brisket and flank samples showing 9.5% and 7.1% occurrence at abattoir, respectively. Neck and rump samples had higher contamination rates of 16.0% and 8.7% at Meat Retailer Outlets. The observed contamination rates among the 31 isolates from raw beef, with brisket and flank showing lower occurrences (9.5% and 7.1%) compared to neck and rump (16.0% and 8.7%), can be attributed to several factors related to the anatomy and handling of the meat. Cuts like the neck and rump may be more susceptible to bacterial contamination due to their proximity to areas with higher microbial loads, such as the gastrointestinal tract, during processing. Additionally, these cuts may undergo different handling practices that increase exposure to contaminants, including greater contact with surfaces or tools that are not adequately sanitized. The brisket and flank, while still at risk, may have less exposure due to their anatomical positioning and the way they are processed, leading to comparatively lower contamination rates.

To lower contamination rates in beef, especially in cuts such as neck and rump that exhibit elevated bacterial levels, various strategies can be applied across the supply chain. Primarily, it is essential to improve hygiene practices during slaughter and processing, which involves thoroughly sanitizing tools and surfaces and ensuring that workers follow stringent personal hygiene protocols [55]. Additionally, the significant difference in Salmonella prevalence between abattoirs and meat retail outlets can be linked to cross‐contamination that occurs during loading and transportation in open vehicles, along with inadequate hygiene practices at retail sites. Factors such as the rehandling of meat, contaminated clothing, and exposure to environmental contaminants like flies and dust further elevate the risks associated with *Salmonella* [10, 56].

Variations in sampling methodologies and environmental conditions also contribute to discrepancies in prevalence rates [11, 19, 49]. A multivariable logistic analysis indicated that meat handlers with less than 1 year of experience were 26 times more likely to contaminate meat with *Salmonella* compared to more experienced handlers. Additionally, those without prior food safety training were nearly six times more prone to contamination [57, 58]. Overall, training is crucial for improving food handlers’ awareness of hygiene and safe handling practices, thereby reducing the risk of *Salmonella* contamination [41, 59].

The study’s finding that the isolation rate of *Salmonella* spp. was 15 times greater in abattoirs and meat retail outlets without recent medical checkups for food handlers underscores the critical importance of health monitoring in preventing foodborne illnesses. This correlation aligns with research by Azanaw et al. [60], Chekol et al. [61], and Teferi et al. [42], which collectively indicate that the lack of regular medical assessments significantly increases the risk of microbial contamination in the food processing chain. Without these health evaluations, asymptomatic carriers of *Salmonella* may remain undetected, facilitating the transmission of pathogens through direct contact with food or surfaces. Furthermore, food handlers who do not undergo routine checkups may lack awareness of proper hygiene practices, leading to unsafe handling and increased contamination risks. This evidence highlights the necessity for implementing mandatory health screenings and training programs for food handlers to enhance food safety protocols and ultimately protect consumer health.

Furthermore, *Salmonella* isolation was 4.29 times higher in outlets where handlers did not use sanitizer for cleaning hands and utensils, supporting Ansari‐Lari et al. [58], who emphasized handwashing as critical for preventing cross‐contamination. The likelihood of *Salmonella* presence was 4.8 times greater when handlers did not wash hands with soap before and after meat handling, consistent with findings from Ntanga et al. [62] and Geresu and Desta [34].

Teferi et al. [42] noted that wearing protective clothing reduces cross‐contamination risks. The current study showed a significant correlation between the absence of protective clothing and higher *Salmonella* isolation rates in raw beef and meat contact surfaces, with six times greater risk for those not wearing protective apparel, corroborating Chepkemoi et al. [63] and Geresu and Desta [34].

Additionally, using the same equipment for meat and offal processing was linked to higher *Salmonella* isolation rates. The study also revealed a significant correlation between *Salmonella* contamination and beef handled by workers collecting money during sales; samples from these workers had nearly nine times greater chance of contamination than those who did not handle money, supporting Teferi et al. [42] and Geresu and Desta [34].

Poor hygiene of slicing materials in meat retail establishments was found to be nearly 14 times more likely to result in *Salmonella* spp. isolation compared to those with fair or good hygiene. Additionally, the absence of fly control in these outlets significantly increased *Salmonella* contamination rates on beef and meat contact surfaces, with a nearly sixfold higher likelihood of contamination in outlets lacking fly control. This finding aligns with the study reported by, which indicates the rehandling of meat, contaminated clothing, and exposure to environmental contaminants like flies and dust, which further elevate the chances of *Salmonella* contamination [10, 56].

The resistance rate of *Salmonella* spp. to tetracycline was notably high at 93.5%, which is in line with previous study finding 83.9% reported by Wabeto et al. [11] and contrasting with lower rates of 9.52%, 40.50%, and 32.1% reported by Alemu et al. [33], Takele et al. [49], and Garedew et al. [56], respectively. This high resistance may be due to the widespread use of tetracycline in veterinary medicine (Xu et al., 2020) [64], leading to increased resistance [65]. The local community’s high usage of this drug, facilitated by its accessibility, further contributes to this issue [34]. The recent research on 208,233 *Salmonella* genomes from 148 countries between 1900 and 2023 to investigate the global occurrence and AMR patterns also reveal that AMR levels vary geographically based on location, source, and serovar [32].

The ampicillin resistance rate of 67.7% observed in this study is lower than the 100% reported by Geresu et al. [10], indicating potential differences in local bacterial populations or antibiotic usage, while still being higher than several other studies, suggesting variability in resistance patterns across different regions or settings. Sensitivity to amoxicillin‐clavulanic acid was recorded at 87.1%, which is lower than the findings of Alemu et al. [33] but higher than other reports from Ethiopia, reflecting regional differences in resistance mechanisms. Additionally, co‐trimoxazole sensitivity at 83.9% and ceftazidime sensitivity at 70.9% were both lower than some previous findings, highlighting ongoing challenges in managing AR and the need for continuous monitoring of resistance trends to inform effective treatment strategies.

The highest susceptibility observed was 100% for both ciprofloxacin and gentamicin. The susceptibility rate for ciprofloxacin in this study aligns with earlier research conducted in Ethiopia, which reported rates between 86% and 100%. Similarly, the susceptibility rate for gentamicin in similar previous studies ranged from 55.4% to 100% [11, 35, 41, 49]. Inconsistent AMR levels across different regions may stem from variations in sample sizes, bacterial strains, and the inappropriate use of antimicrobials in livestock, which increases selection pressure for resistant genes.

The variation in the multidrug resistance rate of *Salmonella* spp. can largely be attributed to the use of antimicrobial agents in food animals, which are often administered as growth promoters, at sub‐therapeutic levels, or as preventative doses. This practice has been shown to foster the emergence of antimicrobial‐resistant strains on farms, thereby heightening the risks to human health associated with the consumption of contaminated meat products (Tan et al., 2019) [32, 66, 67].

The emergence and spread of antibiotic‐resistant bacteria to the human population can be traced back to the use of antibiotics in livestock for both therapeutic purposes and as growth enhancers [68]. The development of multiple antibiotic resistances (MARs) among these isolates poses a significant concern, as it complicates the treatment of invasive salmonellosis. The notable increase in AMR observed in *Salmonella* spp. is likely reflective of their extensive use in both veterinary and public health practices in Ethiopia [35, 66]. This situation underscores the urgent need for stricter regulations on antibiotic use in agriculture and enhanced monitoring of AMR patterns to safeguard public health.

The widespread use of antibiotics in livestock for both treatment and growth promotion is a critical public health concern, particularly in Ethiopia, where it has been linked to the emergence and spread of antibiotic‐resistant bacteria, including Salmonella spp. [68]. This practice not only facilitates the development of MAR among these pathogens but also poses significant challenges for treating invasive salmonellosis in humans, as conventional antibiotics become less effective against these resistant strains [35, 66]. The extensive reliance on antibiotics in the veterinary sector, often driven by limited regulatory oversight and inadequate knowledge among farmers regarding responsible use, exacerbates the problem. As resistant bacteria can be transmitted to humans through contaminated food, direct contact with animals, or environmental exposure, the public health implications are profound, leading to increased morbidity, longer hospital stays, and higher healthcare costs. This situation underscores the urgent need for comprehensive strategies that include improved antibiotic stewardship, enhanced surveillance of resistance patterns, and education initiatives aimed at promoting responsible antibiotic use in both agricultural and healthcare settings.

## 5. Limitations of the Study

Due to resource constraints, we were unable to confirm the *Salmonella* isolates by using molecular techniques. Understanding of specific serotypes prevalent in the region is crucial, especially since only a few are zoonotic and responsible for foodborne illnesses in humans. However, we could not characterize the *Salmonella* isolates at serotype level. These shortcomings impede a thorough understanding of *Salmonella* epidemiology in the current study region and highlight the necessity for more extensive serotyping and molecular confirmation in future studies.

## 6. Conclusions

The study revealed an 8.4% occurrence of *Salmonella* isolates among sampled population, indicating a notable public health concern. Several key risk factors contributing to this occurrence were identified, including insufficient job training, lack of routine medical check‐ups, inadequate hand hygiene, and minimal use of protective clothing, unsanitary slicing equipment, and lack of hygiene of slicing material, and absence of flies control at meat retailer outlets highlighting the necessity for targeted interventions. The isolates demonstrated complete susceptibility to ciprofloxacin and gentamicin, but alarmingly showed a 93.5% resistance to tetracycline, emphasizing the need for effective antibiotic stewardship and monitoring practices in the meat supply chain. Additionally, it is recommended to implementing introduction of training programs for food handlers focused on hygiene practices, the implementation of mandatory health check‐ups for food handlers, the enforcement of personal protective equipment usage, the execution of regular hygiene audits in food establishments, and the initiation of public awareness campaigns regarding safe food handling to mitigate *Salmonella* risks.

**Nomenclature**
AMR: Antimicrobial Resistance
AOR: Adjusted Odds Ratio
CI: Confidence Interval
WCU: Wachemo University

## Author Contributions

Assefa Alemu and Galana Abaya developed the study design. The study was conducted and data were collected by Assefa Alemu, Galana Abaya, and Girma Godebo. Data analysis and manuscript drafting were carried out by Assefa Alemu, Galana Abaya, Girma Godebo, and Abdulhakim Mussema. All authors contributed to critical reviews.

## Funding

This study was conducted without any financial support.

## Disclosure

All the authors approved the final manuscript version.

## Ethics Statement

The protocol received approval from the review committee of Wachemo University, College of Natural and Computational Sciences. All study participants were informed about the research’s purpose and objectives, and each provided written informed consent.

## Consent

Please see the Ethics Statement.

## Conflicts of Interest

The authors declare no conflicts of interest.

## Data Availability

Data available are upon request from the authors.
